# A Comparison of LASSO Regression and Tree-Based Models for Delayed Cerebral Ischemia in Elderly Patients With Subarachnoid Hemorrhage

**DOI:** 10.3389/fneur.2022.791547

**Published:** 2022-03-10

**Authors:** Ping Hu, Yangfan Liu, Yuntao Li, Geng Guo, Zhongzhou Su, Xu Gao, Junhui Chen, Yangzhi Qi, Yang Xu, Tengfeng Yan, Liguo Ye, Qian Sun, Gang Deng, Hongbo Zhang, Qianxue Chen

**Affiliations:** ^1^Department of Neurosurgery, Renmin Hospital of Wuhan University, Wuhan, China; ^2^Department of Neurosurgery, Affiliated Hospital of Panzhihua University, Panzhihua, China; ^3^Department of Neurosurgery, Huzhou Central Hospital, Huzhou, China; ^4^Department of Neurosurgery, First Hospital of Shanxi Medical University, Taiyuan, China; ^5^Department of Neurosurgery, General Hospital of Northern Theater Command, Shenyang, China; ^6^Department of Neurosurgery, The Second Affiliated Hospital of Nanchang University, Nanchang, China

**Keywords:** delayed cerebral ischemia, subarachnoid hemorrhage, aneurysm, LASSO, tree model

## Abstract

**Backgrounds:**

As a most widely used machine learning method, tree-based algorithms have not been applied to predict delayed cerebral ischemia (DCI) in elderly patients with aneurysmal subarachnoid hemorrhage (aSAH). Hence, this study aims to develop the conventional regression and tree-based models and determine which model has better prediction performance for DCI development in hospitalized elderly patients after aSAH.

**Methods:**

This was a multicenter, retrospective, observational cohort study analyzing elderly patients with aSAH aged 60 years and older. We randomly divided the multicentral data into model training and validation cohort in a ratio of 70–30%. One conventional regression and tree-based model, such as least absolute shrinkage and selection operator (LASSO), decision tree (DT), random forest (RF), and eXtreme Gradient Boosting (XGBoost), was developed. Accuracy, sensitivity, specificity, area under the precision-recall curve (AUC-PR), and area under the receiver operating characteristic curve (AUC-ROC) with 95% *CI* were employed to evaluate the model prediction performance. A DeLong test was conducted to calculate the statistical differences among models. Finally, we figured the importance weight of each feature to visualize the contribution on DCI.

**Results:**

There were 111 and 42 patients in the model training and validation cohorts, and 53 cases developed DCI. According to AUC-ROC value in the model internal validation, DT of 0.836 (95% *CI*: 0.747–0.926, *p* = 0.15), RF of 1 (95% *CI*: 1–1, *p* < 0.05), and XGBoost of 0.931 (95% *CI*: 0.885–0.978, *p* = 0.01) outperformed LASSO of 0.793 (95% *CI*: 0.692–0.893). However, the LASSO scored a highest AUC-ROC value of 0.894 (95% *CI*: 0.8–0.989) than DT of 0.764 (95% *CI*: 0.6–0.928, *p* = 0.05), RF of 0.821 (95% *CI*: 0.683–0.959, *p* = 0.27), and XGBoost of 0.865 (95% *CI*: 0.751–0.979, *p* = 0.69) in independent external validation. Moreover, the LASSO had a highest AUC-PR value of 0.681 than DT of 0.615, RF of 0.667, and XGBoost of 0.622 in external validation. In addition, we found that CT values of subarachnoid clots, aneurysm therapy, and white blood cell counts were the most important features for DCI in elderly patients with aSAH.

**Conclusions:**

The LASSO had a superior prediction power than tree-based models in external validation. As a result, we recommend the conventional LASSO regression model to predict DCI in elderly patients with aSAH.

## Introduction

Subarachnoid hemorrhage (SAH) secondary to the ruptured aneurysm is a potentially fatal cerebrovascular disease that mainly occurs in middle-aged patients <60 years (1, 2). However, the number of elderly patients with SAH has been increasing due to improved life expectancy (3). It was reported that the annual incidence of SAH in persons over 70 years of age was estimated to exceed 25/100,000 (4). Delayed cerebral ischemia (DCI) is the most frequent complication after SAH, affecting approximately 30% of patients and often leading to poor neurology outcomes or deterioration of patients' conditions (5, 6). Nevertheless, the prognostic effect maybe worse when DCI occurs in elderly patients during hospitalization (7). The timely accurate prediction of DCI development is critical for the clinical management and prognosis of elderly patients with SAH; hence, a reliable, precise prediction model for early identifying DCI is urgently needed.

The conventional logistic regression (LR) is still the primary method for developing prediction models for clinical disease. Such as the previous studies revealed that independent risk factors were identified *via* LR to further construct models for predicting DCI in patients with SAH (8–12). Yet, the conventional LR method could not fully utilize the clinical data during the developing model process, may contributing to a low prediction power. Machine learning (ML) as a domain of artificial intelligence can solve this limitation, and recent research showed that ML algorithms outperformed traditional statistic modeling approaches (13–15). Meanwhile, tree-based methods have been considered one of the best and most extensively used statistical ML methods for analyzing the complex clinical data. Tree-based models produce high accuracy and ease of interpretation of results (16). For instance, predicting long-term prognostic outcomes after SAH (17–19), mortality analysis after SAH (20), and utility analysis of management strategies after SAH (21). However, after carefully reviewing the literature, we did not find any research using tree-based methods to predict DCI development in the elderly patient population after aneurysmal SAH (aSAH).

Therefore, the purpose of this study is to develop conventional regression and tree-based models and compare which model had better prediction performance for the DCI development in hospitalized elderly patients after aSAH.

## Materials and Methods

### Study Design and Patient Enrollment

**Figure 1:**
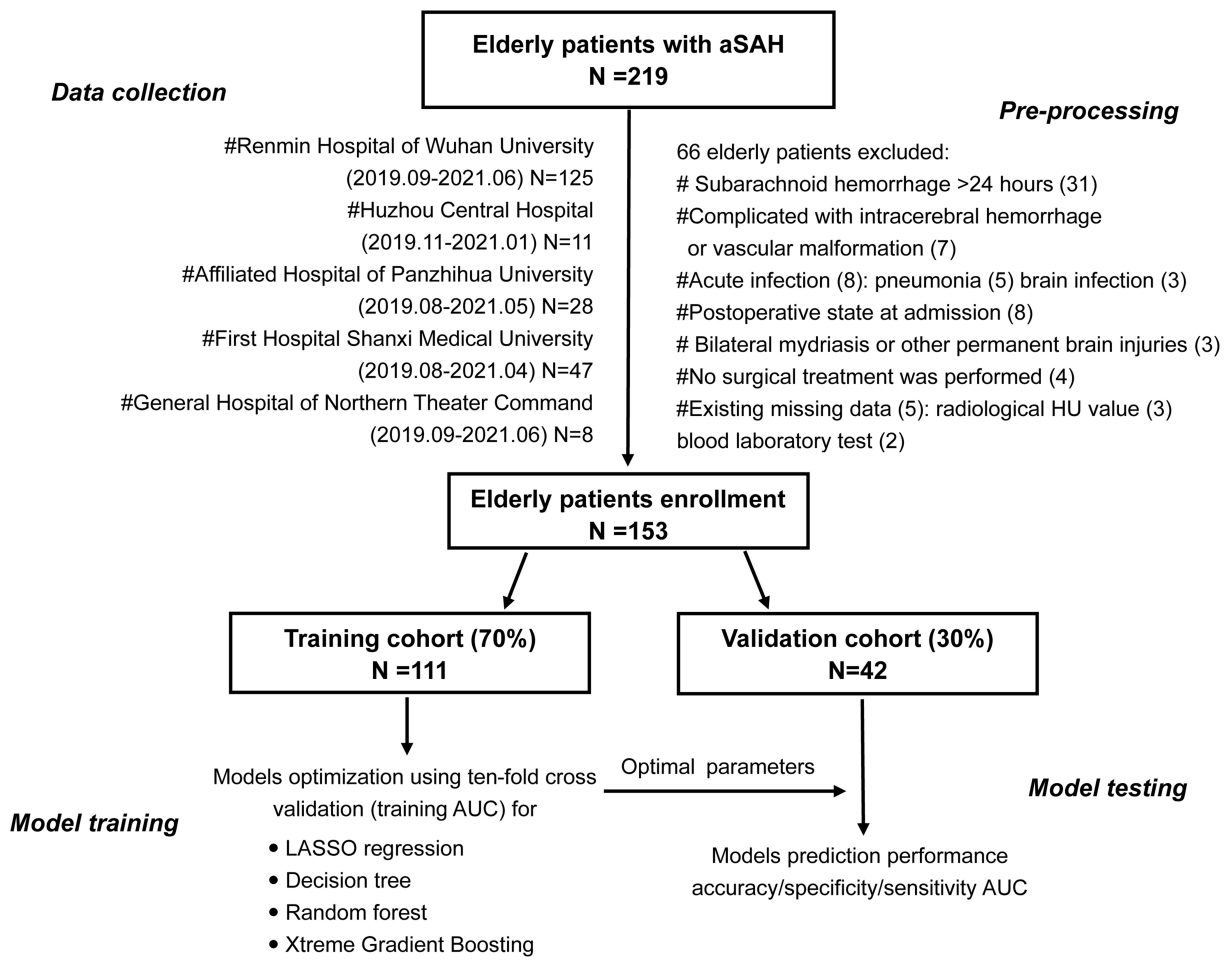
The flowchart that showed exclusion details and the procedure of this study.

This was a multicenter, retrospective, observational cohort study that utilized admission clinical information from the electronic health record system. This study participant consisted of all elderly patients with aSAH within 24 h of onset who were admitted in the department of neurosurgery of several medical centers from April 2019 to June 2021, such as Renmin Hospital of Wuhan University, Huizhou Third People's Hospital, Affiliated Hospital of Panzhihua University, First Hospital of Shanxi Medical University, and General Hospital of Northern Theater Command. The elderly patients were defined as those aged 60 years and older. Out of all consecutive 215 patients, 153 eligible elderly patients with aSAH were eventually enrolled in our study. Figure 1 was a flowchart that showed exclusion details and the procedure of this study. Head CT, head and neck CT angiography, or intracerebral digital subtraction angiography was used for the diagnosis of aSAH.

The inclusion criteria were as follows: elderly patients aged over 60 years, admission within 24 h after onset, spontaneous SAH caused by aneurysm, head CT scan and blood laboratory tests within 24 h after admission, surgical treatment within 3 days after onset, and DCI after SAH occurred within 4–30 days.

The exclusion criteria included aSAH patients complicated with vascular malformation or intracerebral hemorrhage, postoperative state on admission, cases complicated by acute infection, permanent brain injuries or bilateral mydriasis, nonsurgical treatment, and larger missing data.

### Clinical Information Collection

The patient clinical information that included sex, age, past medical history (hypertension, diabetes mellitus, coronary heart disease, smoking, and alcohol consumption), and admission state (World Federation of Neurosurgical Societies [WFNS], Hunt and Hess grade [HH], and modified Fisher scale [mFS]) was collected. In addition, aneurysmal details that included aneurysm location, number, length size, neck size, and surgical treatment method were recorded. Admission blood laboratory tests (D-dimer, glucose, white blood cell [WBC], neutrophil, lymphocyte, and monocyte counts) and CT value of subarachnoid clots and cerebral edema were also utilized in this study. The CT value evaluation method is provided in the Methods in the Data Supplement.

All hospitalized patients received standardized postoperative treatment based on the SAH guidelines (22), such as nimodipine to prevent vasospasms, anti-inflammatory drugs, hemostasis, and analgesic. A postoperative head CT scan was performed to determine the presence of intracranial rebleeding or cerebral infarction.

### Delayed Cerebral Ischemia Definitions

The definition of DCI is consistent with Vergouwen et al. (23). (1) No other cause could have led to the occurrence of a permanent or temporary focal neurological impairment (such as aphasia, apraxia, hemianopia, or neglect) between 4 and 14 days after aSAH; (2) the Glasgow Coma Scale score decreased by at least two points (either on one of its components [eye opening, verbal response, and motor response] or on total score); and (3) head CT scans revealed a low-density area that was not noticeable on admission or immediately after the operation, and there were no other causes except vasospasms between 4 and 30 days after aSAH.

### Sample Size Evaluation

Events per variable with a value of 10 was used to determine the effective sample size in our study (24). A total of six variables were entered into a multivariable regression model in our preliminary analysis. Hence, there should be at least 60 patients with DCI. In addition, according to the incidence rate of 30% of DCI occurrence after SAH worldwide, at least 200 patients should be enrolled in the model training cohort. Based on the limited effective sample size, the least absolute shrinkage and selection operator (LASSO) regression analysis was used to develop a conventional regression model.

### Missing Data Processing

A total of five elderly patients had missing data, which accounted for <5% of the patient population. Therefore, a direct deletion method was applied to process the data (25).

### Prediction Model Development

In this study, each patients with aSAH in the dataset was regarded as a single data point, clinical information measured at admission (demographic data, past medical history, WFNS grade, HH grade, mFS, aneurysm information, treatment methods, serum laboratory test, and image CT value) was used as feature input, and DCI occurred was used as the label of the algorithm. We randomly divided the multicentral data into the model development cohort and model validation cohort in a ratio of 70–30%. The training cohort of 111 patients was used to construct the conventional LASSO regression model and tree models, such as decision tree (DT), random forest (RF), eXtreme Gradient Boosting (XGBoost). We used grid search to find optimal parameters. Since the computational resource is limited, only some critical parameters are taken into account for each model. The searching range and steps of the chosen parameters for all investigated modes are listed below.

### The LASSO Model

The LASSO regression, suitable for small sample size and high-dimensional data, was used to select the most informative prediction variables to construct the model. We used the “glmnet, corrplot, caret” packages of R and five-fold cross-validation to obtain the optimal λ and the variables selecting results.

### The DT Model

Decision tree algorithms partition the sample data by splitting prediction features at discrete cut-points and are usually presented in the form of a tree. The DT algorithm uses the Gini index to determine each split's optimal variable and location in this study. The cost complexity parameter that penalizes more complex trees is used to control the size of the final tree. Several important parameters, such as max_depth, min_samples_split, min_samples_leaf, and max_leaf_nodes were adjusted by the 10-fold cross-validation and “rpart, partykit, caret” packages of R.

### The RF Model

Random forest generates multiple DTs by sampling objects and variables, and then classifies the objects in turn to build a predictive model. Finally, to summarize the classification results of each DT, the mode category in all prediction categories is the category of the RF model prediction object. The important parameters, such as n-estimators, min_sample split, and min_sample_leaf, were determined using the 10-fold cross-validation and “randomForest” package.

### An XGBoostmodel

The XGBoost is an optimized distributed gradient enhancement library whose design is efficient, flexible, and portable. An ML algorithm is implemented under the framework of gradient enhancement. XGBoost provides the promotion of parallel trees, such as gradient boosting decision trees, which can solve many data science problems quickly and accurately. The important parameters, such as gamma, subsample, nrounds, max_depth, eta, colsample_bytree, and min_child_weight were evaluated by “xgboost” package and a 10-fold cross-validation.

### Evaluation of Prediction Model Performances

The area under the receiver operating characteristic (ROC) curve (AUC) with 95% *CI*s, a precision-recall curve, accuracy, sensitivity, and specificity were used to evaluate the model performance. Additionally, we calculated precision and recall indicators using a validation cohort. We used the optimalCutoff function to obtain the optimal threshold of the model outputs to evaluate the model performance. To better demonstrate the generalization of the above-mentioned models, we calculated those indices on both model training and validation cohorts. Furthermore, we compared the errors of the two cohorts to assess the considered models. Finally, to visualize the contribution of each clinical feature, feature importance calculated *via* the XGBoost method was generated to rank their relative influence on the risk of DCI development.

### Statistical Analysis

The Kolmogorov–Smirnov test was used to determine the distribution type of the data before formally analyzing the dataset. Continuous variables analyzed using the Mann–Whitney *U*-test, or independent *t*-test, is presented as a median with interquartile range (IQR) or mean ± SD. Categorical variables analyzed using the chi-square test, or Fisher's exact test are expressed as numbers (percentages). All statistical analyses were two-tailed, and the values of *p* lower than 0.05 were considered statistically significant. All statistical analyses in this study were completed using IBM SPSS Statistics for Windows, version 26.0 (IBM Corp., Armonk NY, USA) and R software (https://www.r-project.org/).

## Results

### Baseline Characteristics

The mean age of elderly patients in the model training and validation cohorts was 67 years (IQR: 63, 71) and 66 years (IQR: 63, 69), respectively. We observed that elderly patients with aSAH were more likely to be women, and there were no significant distinctions in past medical history among the two cohorts. In addition, the admission state, aneurysmal information, admission laboratory results, CT value in subarachnoid clots, and cerebral edema had no significant differences between the two groups. The number of patients with DCI in the two groups was 31 (28%) and 11 (26%). Table 1 shows the baseline characteristics in training and validation cohorts. Moreover, we analyzed the baseline information of elderly patients with or without DCI in the training cohorts. Details are placed in Table 2.

**Table 1:** Baseline characteristics of the elderly patients in model training and validation cohorts.

**Characteristics**	**Training cohort (*n =* 111)**	**Validation cohort (*n =* 42)**	***P*-value**
Demographics			
Age (years)	67 (63, 71)	66 (63, 69)	0.607
Gender (Female)	77 (69)	29 (69)	1.000
Medical history			
Hypertension	65 (59)	19 (45)	0.195
Diabetes	5 (5)	2 (5)	1.000
CHD	6 (5)	2 (5)	1.000
Smoking	20 (18)	7 (17)	1.000
Drinking	16 (14)	4 (10)	0.595
WFNS grade			0.500
I–II	76 (69)	28 (66)	
III	19 (17)	5 (12)	
IV	8 (7)	7 (17)	
V	8 (7)	2 (5)	
Hunt and Hess grade			0.191
I–II	63 (57)	29 (69)	
III	34 (31)	7 (17)	
IV	7 (6)	3 (7)	
V	7 (6)	3 (7)	
Modified Fisher scale			0.925
1–2	47 (42)	17 (41)	
3	33 (30)	11 (26)	
4	31 (28)	14 (33)	
Aneurysm location			0.240
ACA	98 (88)	40 (95)	
PCA	13 (12)	2 (5)	
Aneurysm number			1.000
Single	98 (88)	38 (90)	
Multiple	13 (12)	4 (10)	
Mean aneurysm size
Neck (mm)	3.2 (2.5, 4.25)	3.2 (2.5, 3.98)	0.530
Length (mm)	4.5 (3.5, 5.95)	4.14 (3, 5.8)	0.293
Aneurysm treatment			0.156
Clipping	50 (45)	25 (60)	
Coiling	61 (55)	17 (40)	
Admission laboratory results			
Glucose (mmol/L)	7.3 (6.24, 8.55)	6.94 (5.97, 8.17)	0.506
D-dimer (mg/L)	1.48 (0.78, 3.43)	1.42 (0.88, 3.06)	0.917
WBC (10^∧^9/L)	11.3 (8.7, 13.73)	11.25 (9.69, 13.97)	0.606
Neutrophil (10^∧^9 /L)	9.67 (7.33, 12.22)	9.1 (7.56, 11.53)	0.731
Lymphocyte (10^∧^9 /L)	0.85 (0.66, 1.19)	0.94 (0.69, 1.25)	0.778
Monocytes (10^∧^9 /L)	0.47 (0.33, 0.68)	0.39 (0.32, 0.66)	0.629
Admission CT value (HU)			
ClotCT	57.88 (53, 62.24)	59.41 (52.82, 62)	0.806
EdemaCT	26.4 (24.3, 28.8)	26.22 (22.33, 29.08)	0.589
DCI	31 (28)	11 (26)	0.990

*DCI indicates delayed cerebral ischemia; ACA aneurysm includes anterior cerebral artery, middle cerebral artery, internal cerebral artery, anterior communicating artery; posterior communicating artery; PCA aneurysm includes posterior cerebral artery, basilar artery, anterior inferior cerebellar artery, posterior inferior cerebellar artery, vertebral artery; ACA, anterior circulation aneurysm; PCA, posterior circulation aneurysm; WBC, White blood cell; CT, computer tomography; HU, Hounsfield Unit; WFNS, World Federation of Neurosurgical Surgeons; CHD, Coronary heart disease*.

**Table 2:** Baseline characteristics of model training cohort based on delayed cerebral ischemia.

**Characteristics**	**Total (*n =* 111)**	**Non-DCI (*n =* 80)**	**DCI (*n =* 31)**	***P*-value**
Demographics				
Age (years)	67 (63, 71)	67 (63.75, 70.25)	65 (62, 71)	0.571
Gender (Female)	77 (69)	59 (74)	18 (58)	0.168
Medical history				
Hypertension	65 (59)	50 (62)	15 (48)	0.255
Diabetes	5 (5)	5 (6)	0 (0)	0.319
CHD	6 (5)	2 (2)	4 (13)	0.05
Smoking	20 (18)	12 (15)	8 (26)	0.292
Drinking	16 (14)	10 (12)	6 (19)	0.376
WFNS grade				<0.001
I-II	76 (68)	62 (78)	14 (45)	
III	19 (17)	13 (16)	6 (19)	
IV	8 (7)	3 (4)	5 (16)	
V	8 (7)	2 (2)	6 (19)	
Hunt and Hess grade				0.009
I–II	63 (57)	52 (65)	11 (35)	
III	34 (31)	23 (29)	11 (35)	
IV	7 (6)	3 (4)	4 (13)	
V	7 (6)	2 (2)	5 (16)	
Modified Fisher scale				0.623
1–2	47 (42)	35 (44)	12 (39)	
3	33 (30)	25 (31)	8 (26)	
4	31 (28)	20 (25)	11 (35)	
Aneurysm location				1.000
ACA	98 (88)	70 (88)	28 (90)	
PCA	13 (12)	10 (12)	3 (10)	
Aneurysm number				0.754
Single	98 (88)	71 (89)	27 (87)	
Multiple (≥2)	13 (12)	9 (11)	4 (13)	
Mean aneurysm size				
Neck (mm)	3.2 (2.5, 4.25)	3.2 (2.54, 4.12)	3.4 (2.35, 4.3)	0.95
Length (mm)	4.5 (3.5, 5.95)	4.25 (3.44, 5.53)	4.9 (3.85, 6.25)	0.207
Aneurysm treatment				<0.001
Clipping	50 (45)	26 (32)	24 (77)	
Coiling	61 (55)	54 (68)	7 (23)	
Admission laboratory results				
Glucose (mmol/L)	7.3 (6.24, 8.55)	7.28 (6.15, 8.48)	7.4 (6.36, 8.7)	0.696
D-dimer (mg/L)	1.48 (0.78, 3.43)	1.35 (0.76, 3.08)	2.21 (0.86, 4.39)	0.064
WBC (10^∧^9/L)	11.3 (8.7, 13.73)	10.13 (8.57, 12.7)	13.7 (11.11, 15.2)	0.002
Neutrophil (10^∧^9 /L)	9.67 (7.33, 12.22)	8.34 (7, 11.17)	12.22 (9.37, 13.86)	0.003
Lymphocyte (10^∧^9 /L)	0.85 (0.66, 1.19)	0.92 (0.68, 1.28)	0.77 (0.65, 1.05)	0.293
Monocytes (10^∧^9 /L)	0.47 (0.33, 0.68)	0.46 (0.31, 0.64)	0.5 (0.38, 0.76)	0.206
Admission CT value (HU)				
ClotCT	57.88 (53, 62.24)	56.39 (52.23, 60)	62.59 (57.09, 65.85)	<0.001
EdemaCT	26.4 (24.3, 28.8)	26.66 (24.96, 29)	26 (23.6, 27.08)	0.175

*ACA, anterior circulation aneurysm; PCA, posterior circulation aneurysm; WBC, White blood cell; CT, computer tomography; HU, Hounsfield Unit; WFNS, World Federation of Neurosurgical Surgeons; CHD, Coronary heart disease. ACA aneurysm includes anterior cerebral artery, middle cerebral artery, internal cerebral artery, anterior communicating artery; posterior communicating artery; PCA aneurysm includes posterior cerebral artery, basilar artery, anterior inferior cerebellar artery, posterior inferior cerebellar artery, vertebral artery*.

### LASSO and Tree Models Development

**Figure 2:**
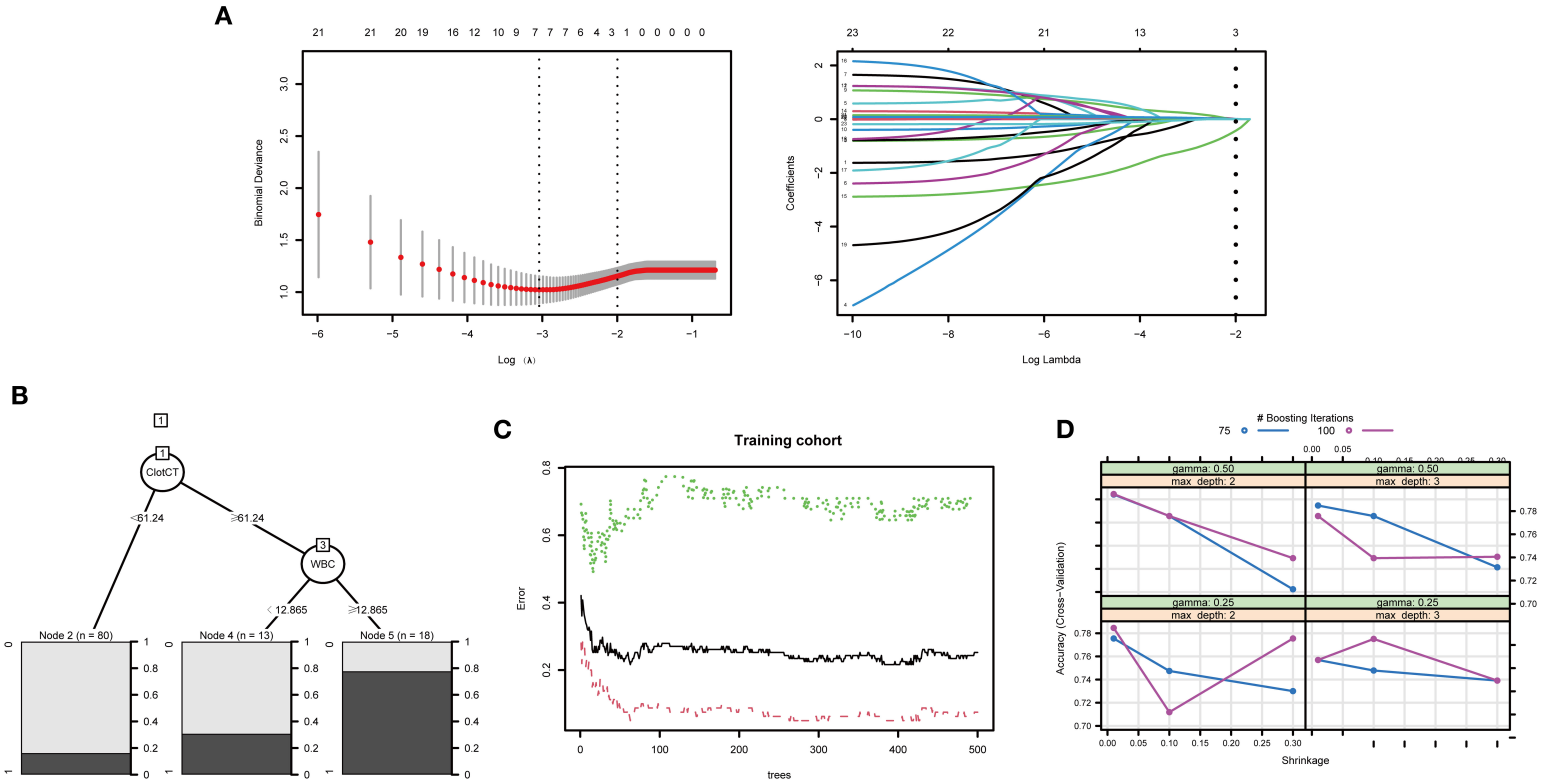
The training process and optimal parameters of the LASSO and tree ML models were demonstrated. **(A)** Demonstrates that 23 features finally decreased to two features when using an optimal λ of 0.1356784 and log(λ) of −1.997 parameters. **(B)** Shows that the optimal decision nodes were CT value of subarachnoid clot and WBC count during the training process of DT. The minimum error was obtained when the optima tree number was 63, the training process of RF is shown in **(C)**. **(D)** Displays the training process of XGBoost, and we can obtain the best prediction power when gamma of 0.25, maximum depth of 2, and nrounds value of 100.

The training process and optimal parameters of the LASSO and tree-based models are demonstrated in Figure 2. In the regression model, we used the LASSO method to select the optimal predictors. An optimal λ of 0.1356784 and log (λ) of −1.997 were adopted in LASSO, and Figure 2A demonstrates that 23 features finally decreased to two features when using the above parameters. The independent predictors were CT value in subarachnoid clots (adjusted odds ration [a*OR*]: 1.115, 95% *CI*: 1.028–1.220, *p* = 0.011) and aneurysm treatment method (a*OR*: 0.196, 95% *CI*: 0.067–0.522, *p* = 0.001) after the multivariable regression analysis. Min_samples_split, min_samples_leaf, and max_leaf_nodes were set to 2, 2, 0, respectively, and Figure 2B shows that the optimal decision nodes were CT values of subarachnoid clot and WBC count during the training process of DT. When n_estimators, min_sample_leaf, and min_sample_split indiceswere set to 63, 4, 2, respectively, and Figure 2C demonstrates that the minimum error was 0.05 corresponding to the optimal tree number of 63 during the training process of RF. Figure 2D displays the training process of XGBoost, and we can obtain the best prediction power when gamma of 0.25, subsample of 0.5, nrounds of 100, max_depth of 2, eta of 0.01, colsample_bytree of 1, and min_child_weight of 1. In addition, the optimal thresholds of LASSO, DT, RF, and XGBoost were 0.3, 0.13, 0.48, and 0.43, respectively.

### LASSO and Tree Models Performance and Validation

**Figure 3:**
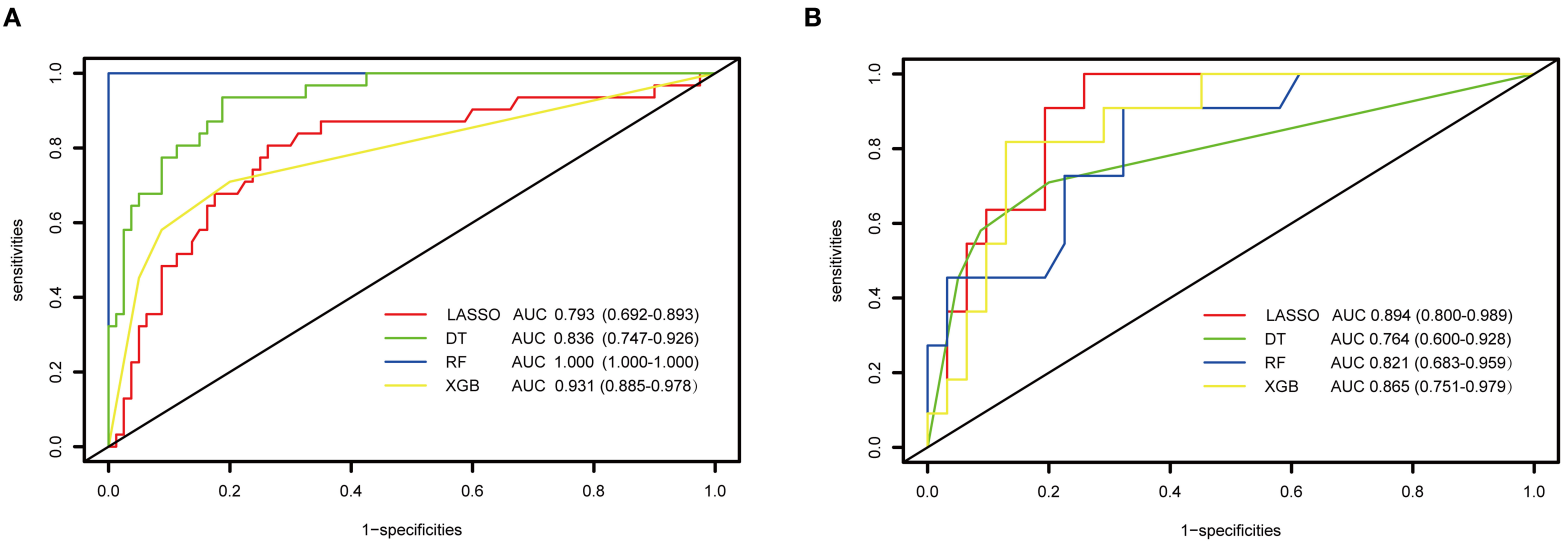
The performance and evaluation of the LASSO regression and tree-based models. **(A)** ROC and AUC value of LASSO and tree-based modes in training cohort; **(B)** the ROC and AUC value of LASSO and tree-based modes in validation cohort. ROC, receiver operating characteristic curve; AUC, area under the curve; LASSO, least absolute shrinkage and selection operator; DT, decision tree; RF, random forest; XGBoost, extreme gradient boosting.

When using the training cohort to evaluate the model performance, the LASSO model had a lowest AUC-ROC value of 0.793 (0.692, 0.893) than the single DT of 0.836 (95% *CI*: 0.747–0.926, *p* = 0.15), RF of 1 (95% *CI*: 1–1, *p* < 0.05), and XGBoost of 0.931 (95% *CI*: 0.885–0.978, *p* = 0.01). Moreover, the accuracy of 80.9% of the LASSO was lower than the RF of 85.7% and the XGBoost of 83.3%. However, the LASSO scored a highest AUC value of 0.894 (95% *CI*: 0.8–0.989) in external verification than DT of 0.764 (95% *CI*: 0.6–0.928, *p* = 0.05), RF of 0.821 (95% *CI*: 0.683–0.959, *p* = 0.27), and XGBoost of 0.865 (95% *CI*: 0.751–0.979, *p* = 0.69). Figure 3 shows the performance and evaluation of the LASSO regression model and tree ML models.

**Figure 4:**
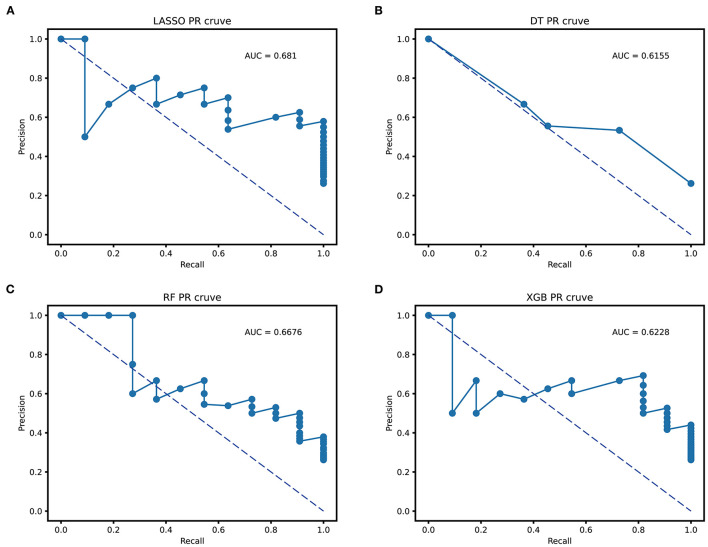
Area under the precision-recall curve (AUC-PR) of all prediction models. **(A)** the AUC-PR of LASSO; **(B)** the AUC-PR of DT; **(C)** the AUC-PR of RF; **(D)** the AUC-PR of XGBoost. LASSO, least absolute shrinkage and selection operator; DT, decision tree; RF, random forest; XGBoost, extreme gradient boosting.

Table 3 illustrates the accuracy, specificity, sensitivity, precision, and recall indicators of the above models. As we can see, the RF model with an accuracy of 100% (100%) is higher than other models using the training cohort. However, its accuracy value decreased to 85.7% (83.7, 100%) in external validation cohort. On the contrary, the XGBoost model with an accuracy value of 87.4 and 83.3% had a stable performance in the two cohorts, and the DT model with an accuracy value of 81.1 and 78.5% performed the worst among all tree models. In the regression model, the LASSO model's accuracy improved by 3.5% from training to the external validation cohort. Moreover, the LASSO model had a higher precision and recall value of 62 and 90% than tree-based models in external validation. When evaluating the model performance using area under the precision-recall curve (AUC-PR), LASSO model scored a highest AUC-PR value of 0.681 than DT of 0.615, RF of 0.667, and XGB of 0.622 in external validation. Figure 4 shows the P–R curve and AUC value of all models.

**Table 3:** LASSO and tree-based model performance and validation.

**Validation**	**Model**	**Accuracy (%)**	**Sensitivity (%)**	**Specificity (%)**	**Precision**	**Recall**
Internal	LASSO	77.4	48.3	88.7	*NA*	*NA*
	DT	81.1	77.7	81.7	*NA*	*NA*
	RF	100	100	100	*NA*	*NA*
	XGBoost	87.4	97.5	61.3	*NA*	*NA*
External	LASSO	80.9	54.5	73.8	62%	90%
	DT	78.5	66.6	80.5	57%	36%
	RF	85.7	100	83.7	62%	45%
	XGBoost	83.3	96.7	45.4	57%	36%

*LASSO indicates least absolute shrinkage and selection operator; DT, decision tree model; RF, random forest; XGBoost, extreme gradient boosting*.

As shown in the Supplementary Table 1, the errors of LASSO, DT, RF, and XGBoost in model training were 18.1, 20.5, 21.6, and 19.8%, respectively, while in the model validation cohort, the error percentage were 23.7, 26.9, 26.1, and 21.2%, respectively.

### Feature Importance

**Figure 5:**
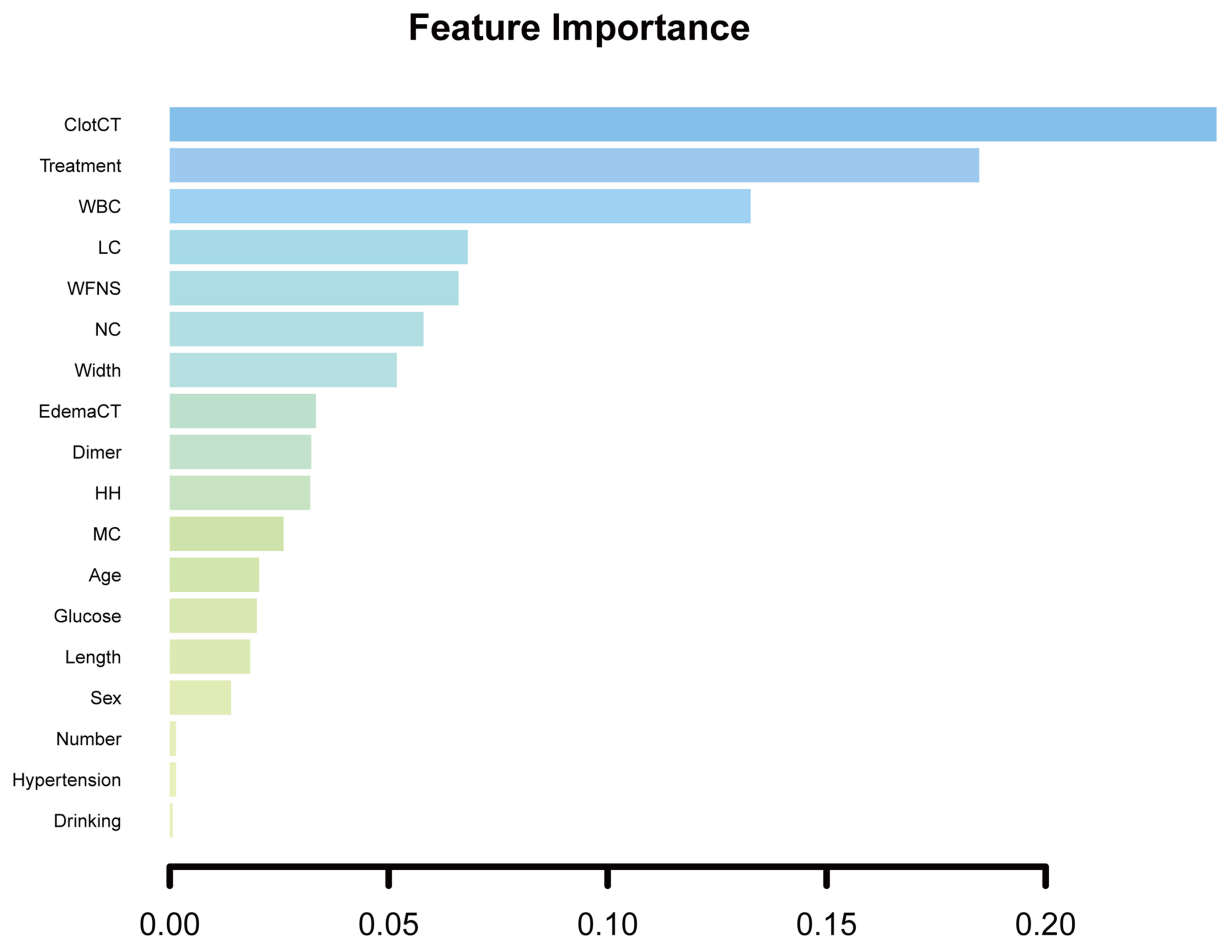
All admission clinical feature importance. ClotCT, CT value of subarachnoid blood clot; WBC, white blood cell; LC, lymphocyte; NC, neutrophil; edemaCT, CT value of cerebral edema; HH, Hunt and Hess grade; MC, monocyte.

The feature importance was scaled so that the sum added up to 1, with a higher importance score indicating a stronger impact on the occurrence of DCI. The most important three features for DCI prediction in elderly patients were CT value pf subarachnoid clots (0.239), aneurysm therapy methods (0.184), and admission WBC counts (0.132). Figure 5 shows all admission clinical feature importance.

## Discussion

In this study, we enrolled the eligible elderly patients with aSAH from five medical centers and randomly divided them into model training and external validation cohorts, and discussed whether tree-based models can improve the DCI prediction power compared with the regression model during hospitalization. Due to our limited effective sample size, the LASSO method was applied to construct one conventional regression model, and compared with three tree models. To our knowledge, this study is the first to develop tree-based models using complete admission clinical information and to systematically compare the performances of the LASSO regression model for DCI prediction in elderly patients.

The LASSO regression as a special method performs well when reducing data dimensions and multicollinearity among features. For instance, our previous study (26) used the LASSO method to select three optimal variables for establishing a dynamic nomogram for predicting an unfavorable prognosis after aSAH in the case of limited effective sample size. In this study, the optimal independent risk factor for DCI prediction in elderly patients were CT value of subarachnoid clots and aneurysmal treatment method. Previous studies have shown that a CT value of SAH more than 49.95 HU is correlated with DCI. The CT values in SAH are generally considered to represent the density of subarachnoid clots (27). It can reflect the neural inflammatory response after SAH, while the neurovascular inflammation would be a potential mechanism of early brain injury and delay cerebral vasospasm (28, 29). In our study, a CT value > 61.24 HU would be an independent predictor for DCI in elderly patients with aSAH. In addition, our study suggests that an aneurysm endovascular therapy is a vital factor for DCI prevention in elderly patients compared with the neurosurgical treatment. Montalverne et al. (30) reported that endovascular treatment should be considered as a first option for the ruptured aneurysm in elderly patients since an overall favorable prognosis can be achieved in most persons. Yue et al. (31) considered that an interventional treatment presented a better outcome than the surgical treatment for elderly patients. By fitting the above two variables, the AUC and accuracy index of the LASSO regression model demonstrated a good predictive performance, which is generally better than the tree models.

Tree learning algorithms are the most widely used supervised learning methods in clinical making-decision at present (16). In this research, we, respectively, constructed single tree, RF, and XGBoost models for DCI prediction in elderly patients, and the results indicated that classification power of the DT model was worse than other tree models. Although there were no previous research utilizing DT for predicting DCI in elderly patient population; however, Churpek et al. (32) argued that the DT model was still less capable of predicting ward deterioration than the RF and XGBoost models. We know that single tree models, while easy to construct and interpret, do not have much prediction power necessary for our attempts to solve this particular kind of outcome classification problem (33). As we can see, the RF and XGBoost models both improved the prediction ability for DCI based on a single tree model. On the whole, the XGBoost model was even better and stable when applying to training and validation cohorts. The possible explanation is that the expected result of the XGB technology is to build a series of trees, the latter trees can improve the shortcomings of the previous trees, and ultimately reduce the deviation and classification to achieve the best prediction performance (34).

In the field of predicting DCI in hospitalized elderly patients, no research has been performed comparing conventional regression and tree-based methods. Most previous studies aimed to predict the occurrence of unfavorable prognoses among the population of SAH based on the DT method. For example, a prospective cohort study of negative outcomes after aSAH by Liu et al. (11) compared the conventional regression model to the DT model. They found that the DT model had a similar predictive performance to the regression model since both achieved a high accuracy of 0.895 in the validation dataset. In addition, similar literature has been reported the field (17, 19, 35). In our study, although the prediction power of tree models is generally superior to the LASSO in the training cohort; however, the AUC value of LASSO regression was higher than tree models in external validation. Since the prediction ability of the LASSO model varies greatly between two datasets, the small sample size of the validation set may explain this phenomenon.

To visualize the contribution of each feature to the occurrence of DCI, we also calculated the importance of each feature. We can see the CT value of subarachnoid clots, aneurysm therapy methods, and WBC counts that were the three most critical features for DCI prediction in hospitalized elderly patients with aSAH. Why the first two features are most important in predicting the occurrence of DCI in elderly patients has been explained above. The number of WBCs in the peripheral blood is well-known to directly reflect the level of inflammation in the body. A large prospective observational cohort study by Al-Mufti et al. (36) considered that WBCs of more than 12.1 × 10^9^/L were the most important predictor for DCI prediction in patients with good-grade after aSAH. In our study, the WBCs >12.8 × 10^9^/L in the peripheral blood, similar to the previous study's result, was deemed as an important factor for DCI prediction in hospitalized elderly patients. Ruptured aneurysms events in elderly patients often result in poor-grade at admission. Clinical signs of the early pro-inflammatory cytokine cascade caused by aSAH are almost double in poor-grade patients (36, 37). This may interpret the difference in WBC counts between our results and the previous study.

Our research has several points of clinical value. For elderly patients with aSAH as a particular cohort, there is currently no literature on the early prediction of DCI in elderly patients during hospitalization. Based on this, we created and compared the regression and tree models to predict the DCI performance of elderly patients with aSAH. Although DT, RF, and XGBoost had a better prediction performance than the LASSO regression in the training cohort. However, the LASSO model demonstrated a superior generalization ability than all tree-based models in external validation cohort. These results need to be further validated in the future. Second, we found the three most important clinical predictive features: CT value of subarachnoid clots, WBCs in the peripheral blood, and aneurysmal therapy method.

However, the limitation of this study deserves attention. The object of this research was a special elderly population with aSAH, and the time of primary SAH must be guaranteed within 24 h. This has led to the fact that although we have carried out a multi-center study, the sample size was relatively limited. In the future, large-sample prospective clinical studies are still needed to verify our results. Second, the CT value of the subarachnoid blood clot is measured by manually drawing on the ROI, and we should pay attention to the measurement error. Therefore, after agreeing with the radiologist, the CT value was obtained by two clinicians without knowing the patient's clinical information to reduce errors.

## Conclusions

For the early prediction of DCI in elderly patients with aSAH, the LASSO model had a superior prediction power than tree-based models in external validation. As a result, we recommend the conventional LASSO regression model to predict DCI in elderly patients with aSAH. However, these results need to be further validated in the future.

## Data Availability Statement

The raw data the supporting the findings of this study are available from the corresponding author upon reasonable request.

## Ethics Statement

The studies involving human participants were reviewed and approved by the Medical Ethics Committees of five Medical Centers, including Renmin Hospital of Wuhan University (Principal Affiliation Center; WDRM2021-K022), First Hospital of Shanxi Medical University (2021-Y6), Affiliated Hospital of Panzhihua University (202105002), Huzhou Central Hospital (202108005-01), and the General Hospital of Northern Theater Command (Y2021060). Written informed consent for participation was not required for this study in accordance with the national legislation and the institutional requirements.

## Author Contributions

PH and QC: study design. PH, YLi, TY, and QS: literature search. PH, HZ, YLiu, GG, ZS, and XG: data acquisition. PH, YQ, LY, YX, YLiu, and JC: data analysis and statistical analysis. PH, YLi, TY, GD, YLiu, and QC: manuscript preparation, editing, and review. All authors read and approved the final manuscript.

## Funding

This work was supported by the National Natural Science Foundation of China (No. 82001311).

## Conflict of Interest

The authors declare that the research was conducted in the absence of any commercial or financial relationships that could be construed as a potential conflict of interest.

## Publisher's Note

All claims expressed in this article are solely those of the authors and do not necessarily represent those of their affiliated organizations, or those of the publisher, the editors and the reviewers. Any product that may be evaluated in this article, or claim that may be made by its manufacturer, is not guaranteed or endorsed by the publisher.
