# Supplementary material for: A Comparison of LASSO Regression and Tree-Based Models for Delayed Cerebral Ischemia in Elderly Patients With Subarachnoid Hemorrhage
Source: Front Neurol. 2022 Mar 10;13:791547. doi: 10.3389/fneur.2022.791547 (PMC8960268; doi:10.3389/fneur.2022.791547)
Supplement: Supplementary file 1 [file Table_1.docx]

**Table I. The errors of LASSO, DT, RF, XGBoost in model training and validation cohorts**

| **Model (error)** | **training** | **validation** |
| --- | --- | --- |
| LASSO | 18.1% | 23.7% |
| DT | 20.5% | 26.9% |
| RF | 21.6% | 26.1% |
| XGBoost | 19.8% | 21.2% |

LASSO, least absolute shrinkage and selection operator; DT, decision tree; RF, random forest; XGBoost, eXtreme Gradient Boosting.
